# Supplementary material for: Tumor Cell-Specific Retention and Photodynamic Action of Erlotinib-Pyropheophorbide Conjugates
Source: Int J Mol Sci. 2022 Sep 21;23(19):11081. doi: 10.3390/ijms231911081 (PMC9569946; doi:10.3390/ijms231911081)
Supplement: Supplementary file 1 [file ijms-23-11081-s001.zip › ijms-1908827-supplementary.pdf]

Tumor Cell-Specific Retention and Photodynamic Action of

Erlotinib-Pyropheophorbide Conjugates

Erin C. Tracy[1], Ravindra R. Cheruku[2], Ravindra K Pandey[2,*] and Heinz Baumann[1,*]

Supplementary Material:

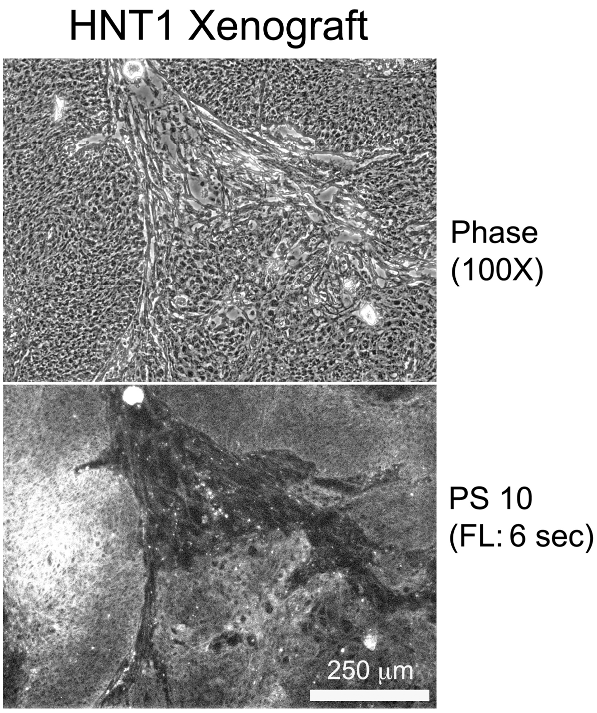

Supplementary Figure S1. SCID mouse bearing a subcutaneous HNT1 xenograft was injected with PS 10 (3 µmol/kg) and 24h later the level of PS 10 fluorescence in the tumor cryosection imaged by fluorescence microscopy. Cellular structure of the tissue section was recorded by phase microscopy.
